# Selective inhibition of protein secretion by abrogating receptor–coat interactions during ER export

**DOI:** 10.1073/pnas.2202080119

**Published:** 2022-07-28

**Authors:** Natalia Gomez-Navarro, Julija Maldutyte, Kristina Poljak, Sew-Yeu Peak-Chew, Jonathon Orme, Brittany J. Bisnett, Caitlin H. Lamb, Michael Boyce, Davide Gianni, Elizabeth A. Miller

**Affiliations:** ^a^Cell Biology Division, Medical Research Council Laboratory of Molecular Biology, Cambridge, CB2 0QH, United Kingdom;; ^b^Discovery Sciences, BioPharmaceuticals R&D, AstraZeneca, Cambridge, CB2 0AA, United Kingdom;; ^c^Department of Biochemistry, Duke University School of Medicine, Durham, NC27710, USA

**Keywords:** COPII vesicles, membrane traffic, ER export

## Abstract

Protein secretion is an essential process that drives cell growth, movement, and communication. Protein traffic within the secretory pathway occurs via transport intermediates that bud from one compartment and fuse with a downstream compartment to deliver their contents. Here, we explore the possibility that protein secretion can be selectively inhibited by perturbing protein–protein interactions that drive capture into transport vesicles. Human proprotein convertase subtilisin/kexin type 9 (PCSK9) is a determinant of cholesterol metabolism whose secretion is mediated by a specific cargo adaptor protein, SEC24A. We map a series of protein–protein interactions between PCSK9, its endoplasmic reticulum (ER) export receptor SURF4, and SEC24A that mediate secretion of PCSK9. We show that the interaction between SURF4 and SEC24A can be inhibited by 4-phenylbutyrate (4-PBA), a small molecule that occludes a cargo-binding domain of SEC24. This inhibition reduces secretion of PCSK9 and additional SURF4 clients that we identify by mass spectrometry, leaving other secreted cargoes unaffected. We propose that selective small-molecule inhibition of cargo recognition by SEC24 is a potential therapeutic intervention for atherosclerosis and other diseases that are modulated by secreted proteins.

## Significance

This study investigates the fundamental cellular process of protein secretion, focusing on the selectivity of this process and how it can be modulated by small-molecule inhibition. We map the protein–protein interaction network that drives secretion via the endoplasmic reticulum (ER) export receptor SURF4. We show that interaction with the COPII cargo adaptor, SEC24, is mediated by a binding pocket that can be occluded by a small-molecule inhibitor. Together, our findings support the exciting prospect that secretion of select proteins can be modulated by inhibition of the protein–protein interaction network that promotes capture into COPII vesicles.

Approximately a third of human protein-coding genes encode proteins that enter the secretory pathway. Such secretory proteins include integral membrane proteins, soluble proteins located in the lumen of membrane-bound organelles, and proteins that are transported outside of the cell. The vast majority of secretory proteins enter the pathway at the endoplasmic reticulum (ER), where they reach their mature state after acquiring posttranslational modifications and interacting with the abundant chaperones of the ER lumen. Secretion-competent mature proteins are exported from the ER via vesicles generated by the COPII (coat protein complex II) coat and delivered to the Golgi apparatus, where they are further modified (1). Vesicle biogenesis occurs via self-assembly of the COPII coat at ER exit sites (ERESs). Protein capture into COPII vesicles occurs via two mechanisms: signal-mediated export and bulk flow (2). In signal-mediated export, specific signals within cargo proteins are recognized by the COPII coat, which then concentrates cargo at sites of vesicle formation. The coat–cargo interaction is direct for many transmembrane proteins, whereas soluble proteins or proteins that do not expose cytoplasmic signals require export receptors that bridge the interaction between cargo and coat (3). In contrast, bulk flow export occurs in the absence of signals and relies on diffusion of a cargo molecule into an ERES followed by passive uptake into a nascent vesicle (4, 5). The extent to which signal-mediated versus bulk flow mechanisms are used for the entire secretome remains to be fully determined.

The COPII coat subunit, Sec24, is the cargo adaptor that confers selective cargo capture and enrichment during vesicle formation. Mutagenesis and structural studies have revealed multiple cargo-binding sites on Sec24 (6–8), which permits recognition of diverse cargoes by a single coat subunit (3). The repertoire of cargo molecules recognized by the coat is further expanded by multiple isoforms of Sec24: *Saccharomyces cerevisiae* express three Sec24 homologs (Sec24, Iss1/Sfb2, and Lst1/Sfb3); humans express four isoforms (SEC24A–D). SEC24A and SEC24B share 60% sequence identity, and SEC24C and SEC24D similarly share more than 50% sequence identity. Accordingly, the four isoforms can be classified into two subfamilies, SEC24A/B and SEC24C/D, that share nonoverlapping recognition specificity (9–12).

The specificity of Sec24-mediated cargo recognition raises the possibility that secretion, an essential process, might be selectively targeted by preventing a subset of Sec24–cargo interactions. Indeed, release of the secreted proprotein convertase subtilisin/kexin type 9 (PCSK9) was selectively perturbed in SEC24A knockout(KO) mice (13). Because PCSK9 participates in cholesterol homeostasis, the predominant physiological effect of SEC24A KO was reduced circulating cholesterol, suggesting a novel therapeutic avenue for cardiovascular health. Here, we sought to probe the network of protein–protein interactions that contribute to PCSK9 secretion with the goal of understanding which interactions might be perturbed to achieve a therapeutic outcome.

PCSK9 secretion via SEC24A is facilitated by the ER export receptor SURF4, which also mediates secretion of multiple calcium-binding extracellular matrix proteins that employ N-terminal ϕ-P-ϕ signals, where ϕ is any hydrophobic amino acid (14, 15). This motif has been termed the ER-ESCAPE (ER-Exit by Soluble Cargo using Amino-terminal Peptide-Encoding) motif (15). The precise mechanism by which SURF4 recognizes this signal remains unknown, and the nature of the interaction between SURF4 and SEC24A also remains unclear. Here, we demonstrate that the SEC24A–SURF4 interaction is driven by a conserved cargo-binding domain on SEC24A known as the B-site (7, 8), which can be occluded by small-molecule treatment (16) to selectively inhibit secretion of PCSK9. Moreover, we identify additional clients of SURF4 that, like PCSK9, use N-terminal ER-ESCAPE motifs to achieve SURF4-dependent secretion. The network of client–receptor–adaptor interactions revealed by our study lends support to the concept of selective inhibition of protein secretion as a therapeutic target.

## Results

### PCSK9 Secretion Requires Both SEC24A and SURF4.

**Fig. 1.**
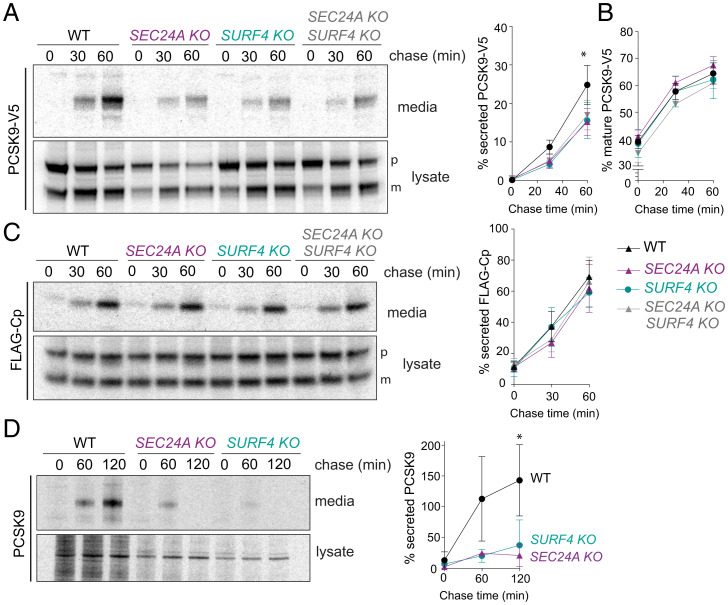
PCSK9 secretion requires SEC24A and SURF4. (*A*) PCSK9-V5 maturation and secretion were examined in WT and KO cell lines by pulse–chase with [^35^S]methionine. PCSK9-V5 was immunoprecipitated with α-V5 from lysates and conditioned media at the indicated times and detected by SDS-PAGE and autoradiography. Percentage of secreted PCSK9 was calculated as [band intensity in media at a given time point]/[total protein intensity (media + lysate) at time 0]. PCSK9 is detected as proprotein (p) and mature (m) bands. Plots show the mean ± SD of three independent experiments. Statistical test was a one-way ANOVA with Dunnett’s correction for multiple comparisons, *P < 0.05. (*B*) Percentage of mature PCSK9 was calculated as [band intensity of the lower MW (mature) species at a given time point]/[total protein (mature + proprotein + secreted) at time 0]. Plots show the mean ± SD of three independent experiments. Statistical test was a one-way ANOVA with Dunnett’s correction for multiple comparisons; no significant difference was detected. (*C*) FLAG-Cp secretion was examined in WT and KO cell lines. FLAG-Cp was immunoprecipitated with α-FLAG from lysates and conditioned media at the indicated times and detected by SDS-PAGE and autoradiography. Autoradiographs are representative of three independent experiments. As in *A*, secretion of FLAG-Cp was quantified by phosphorimage analysis. FLAG-Cp is detected as precursor (p) and mature (m) bands. Plots of the mean ± SD are shown in the right-hand panel. (*D*) Endogenous PCSK9 secretion from the indicated HuH7 cell lines was quantified by pulse–chase with [^35^S]methionine. PCSK9 was immunoprecipitated with α-PCSK9 from lysates and conditioned media at the indicated times and detected by SDS-PAGE and autoradiography. Plots of the mean ± SD of three independent experiments are shown in the right-hand panel, *P < 0.05. PAGE, polyacrylamide gel electrophoresis.

Given the previously described roles for SEC24A and SURF4 in PCSK9 secretion (13, 14), we first evaluated the effects of deletion of these export factors on PCSK9 secretion in TREx-293 cells heterologously expressing V5-tagged (PCSK9-V5). We performed pulse–chase experiments to compare the kinetics of secretion in wild-type (WT) and KO cell lines. As expected, deletion of either SEC24A or SURF4 resulted in decreased release of PCSK9-V5 to the media, with no additive effect observed in the double KO (Fig. 1*A*). Importantly, proteolytic processing of PCSK9-V5, which is required for secretion competence, remained unperturbed in the KO lines (Fig. 1*A* and *B*). Secretion defects were rescued by reintroduction of SEC24A and SURF4 complementary DNAs (cDNAs) by transient transfection (*SI Appendix,* Fig. S1*A* and *B*). We also tested PCSK9-V5 secretion in KO lines deleted for the remaining SEC24 isoforms, SEC24B, SEC24C, and SEC24D, finding that only SEC24A KO reduced PCSK9-V5 secretion (*SI Appendix,* Fig. S1*C* and *D*). These findings differ from previous results that used steady-state immunoblotting to test PCKS9 secretion in small interfering RNA (siRNA) knockdown conditions, where depletion of SEC24B and SEC24C also perturbed PCSK9 secretion (17).

We measured global secretion using pulse–chase analysis of a FLAG-tagged bulk flow marker, the C‐terminal protease domain (Cp) of the capsid protein of Semliki Forest virus (4). FLAG-Cp was secreted normally in both SEC24A KO and SURF4 KO cell lines, suggesting bulk secretion remained intact (Fig. 1*C*). Finally, KO of either SEC24A or SURF4 in the hepatic cell line HuH7, which endogenously expresses PCSK9, resulted in an even more dramatic reduction in PCSK9 secretion (Fig. 1*D*). The magnitude of this effect suggests that in cells specialized for PCSK9 secretion, both SEC24A and SURF4 play essential functions in PCSK9 ER export. We note that secreted PCSK9 decreased in abundance at later time points in the KO cell lines, perhaps due to increased degradation that is masked when PCSK9 levels are higher.

### SURF4 Interacts with the B-Site of SEC24.

**Fig. 2.**
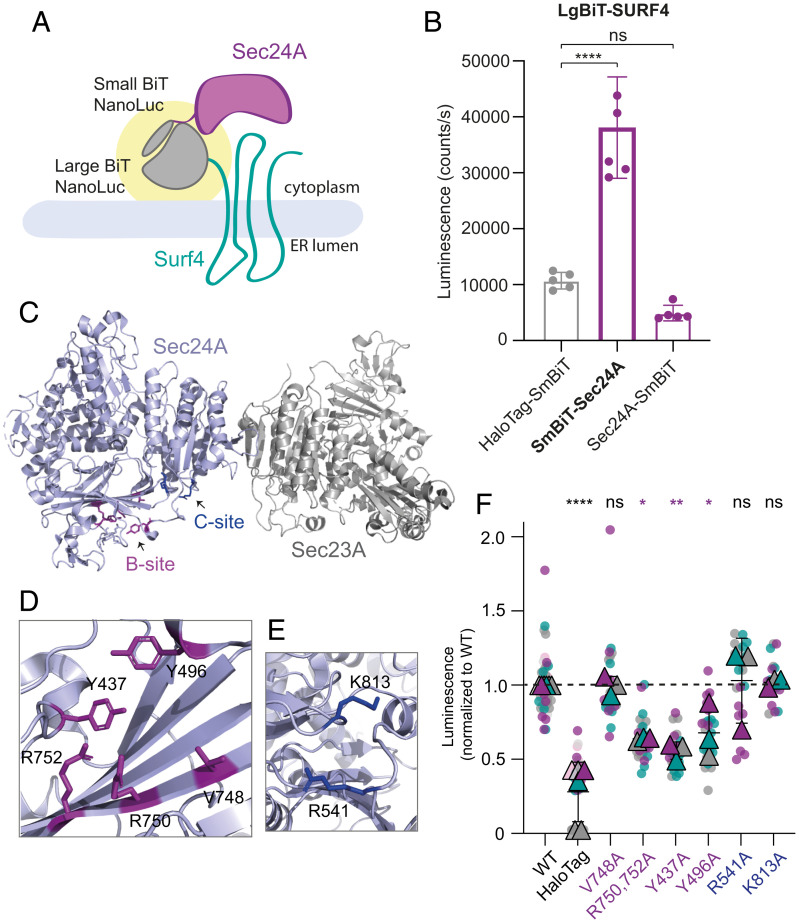
SURF4 and SEC24A interact via the B-site of Sec24. (*A*) Cartoon of the NanoBiT complementation assay for measuring SEC24A and SURF4 interaction, where complex formation results in a reconstituted luminescence signal (yellow halo). (*B*) Cellular luminescence was measured for the indicated NanoBiT constructs expressed in SEC24A SURF4 double KO cells. Graph shows the mean luminescence ± SD (*n* = 6); statistical test was a one-way ANOVA with Dunnett’s correction for multiple comparisons, ****P < 0.0001. (*C*) Crystal structure of the SEC24A–SEC23A complex (Protein Data Bank accession No. 5VNO). Residues in the B-site are shown in purple, and residues in the C-site are shown in blue. (*D* and *E*) Structural detail of the B- and C-sites, respectively, with key residues shown as sticks. (*F*) Luminescence values measured upon coexpression of LgBiT-SURF4 with the indicated SmBiT-SEC24A mutants normalized to WT values. Data correspond to six technical replicates per biological replicate and three independent experiments. The plotted triangles represent the mean ± SD of the three biological replicates. Statistical test was a one-way ANOVA with Dunnett’s correction for multiple comparisons, *P < 0.05, **P < 0.01, ****P < 0.0001. ns, not significant.

In order to characterize the mechanisms of selective SEC24A- and SURF4-mediated secretion, we sought to characterize the interaction between SURF4 and SEC24A. We employed a cell-based protein–protein interaction assay based on NanoBiT (NanoLuc Binary Technology) enzymatic complementation technology. We fused the two fragments of NanoLuc, Large BiT (LgBiT, 18 kDa), and Small BiT (SmBiT, 11 amino acids), to the N- and C-termini of SEC24A and SURF4 and assayed luminescence complementation of the paired tagged fusions (Fig. 2*A*). The different linkage orientations revealed optimal signal with SmBiT appended to the N-terminus of SEC24A and LgBiT appended to the N-terminus of SURF4 (Fig. 2*A*). Luminescence from the SEC24A-SURF4 pair was fourfold higher than luminescence for a control interaction that paired LgBiT-SURF4 with cytoplasmic HaloTag-SmBiT control (Fig. 2*B*). Tagging of either SEC24A or SURF4 at the C terminus resulted in lower levels of luciferase activity (Fig. 2*B* and *SI Appendix,* Fig. S2*A*). To demonstrate the specificity of the interaction, we measured the luminescent signal upon increasing expression of untagged SURF4, which indeed decreased luminescence, presumably by competing for interaction with SmBiT-SEC24A (*SI Appendix,* Fig. S2*B*).

We next mutated surface-exposed residues on SEC24A previously described as cargo-binding sites (6–8) and screened for mutants that reduced the reconstituted luminescence signal of the SEC24A-SURF4 NanoBiT pair. We targeted two well-characterized cargo interaction sites, the B- and C-sites (Fig. 2*C*), making multiple mutations in each region, none of which perturbed protein stability (Fig. 2*D* and *E* and *SI Appendix,* Fig. S2*C*). Mutations in the C-site (R541 and K813) did not perturb the interaction between SEC24A and SURF4; however, mutation of four residues that line a conserved pocket comprising the B-site (R750, R752, Y437, and Y496) significantly reduced the luminescence signal, suggesting impaired interaction between SEC24A and SURF4 (Fig. 2*F*).

### SURF4–SEC24A Interaction Is Disrupted by 4-phenylbutyrate (4-PBA).

**Fig. 3.**
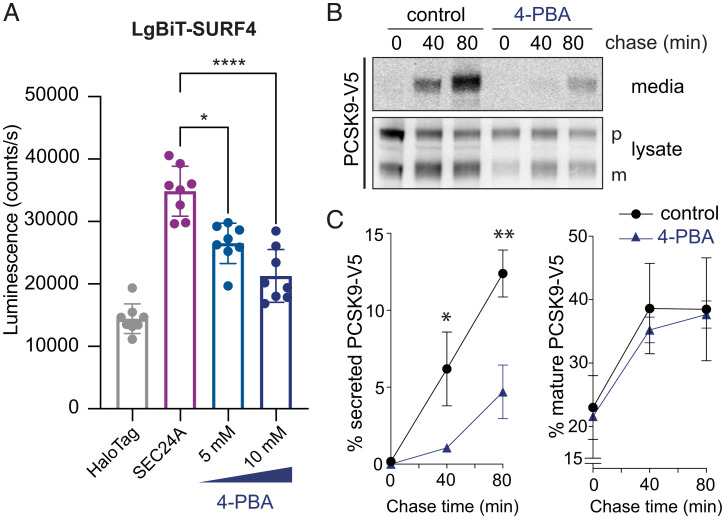
4-PBA reduces the interaction between SEC24A and SURF4 and impairs PCSK9 secretion. (*A*) Luminescence that reports on the interaction between SURF4 and SEC24A was measured in the presence of the indicated concentrations of 4-PBA. The NanoBiT reporters were induced overnight, and cells were treated with 4-PBA for 4 h. The graph shows the mean luminescence ± SD (*n* = 8); statistical test was a one-way ANOVA with Dunnett’s correction for multiple comparisons, *P < 0.05, ****P < 0.0001. (*B*) PCSK9-V5 maturation and secretion was examined in the presence of 10 mM 4-PBA, which was added during the starvation phase of a pulse–chase experiment and maintained over the course of the experiment. PCSK9-V5 was immunoprecipitated with α-V5 from lysates and the conditioned media at the indicated times and detected by SDS-PAGE and autoradiography. PCSK9 is detected as proprotein (p) and mature (m) bands. (*C*) PCSK9 secretion into the media (left plot) and maturation (right plot) were quantified by phosphorimage analysis as described for Fig. 1. Plots show the mean ± SD of three independent experiments. Statistical test was an unpaired *t* test, *P < 0.05, **P < 0.01. PAGE, polyacrylamide gel electrophoresis.

Having identified the SEC24A B-site as driving the interaction between SEC24A and SURF4, we tested whether occlusion of the B-site by 4-PBA (16) perturbs interaction with SURF4. Indeed, in our NanoBiT assay, increasing concentrations of 4-PBA resulted in a reduction in luminescence, suggesting that 4-PBA competes with SURF4 at the B-site (Fig. 3*A*). 4-PBA at those concentrations did not affect cell viability (*SI Appendix,* Fig. S3*A*). We next asked whether impaired SEC24A–SURF4 interaction caused by 4-PBA resulted in deficient trafficking of SURF4-dependent cargo proteins. We performed pulse–chase experiments to measure PCSK9 secretion in the presence of 10 mM 4-PBA (Fig. 3*B*). Indeed, 4-PBA significantly reduced PCSK9 secretion while not affecting protein maturation (Fig. 3*C*), suggesting that PCSK9 secretion indeed relies on the B-site of SEC24 for its ER export. We also analyzed the effects of 5-phenylvaleric acid (5-PVA), a 4-PBA analogue that has a similar affinity for Sec24 (16), and observed a comparable reduction in the SEC24A–SURF4 interaction (*SI Appendix,* Fig. S3*B*).

### In vivo Proteomic Analysis of SURF4 KO Cells Reveals Additional Clients.

**Fig. 4.**
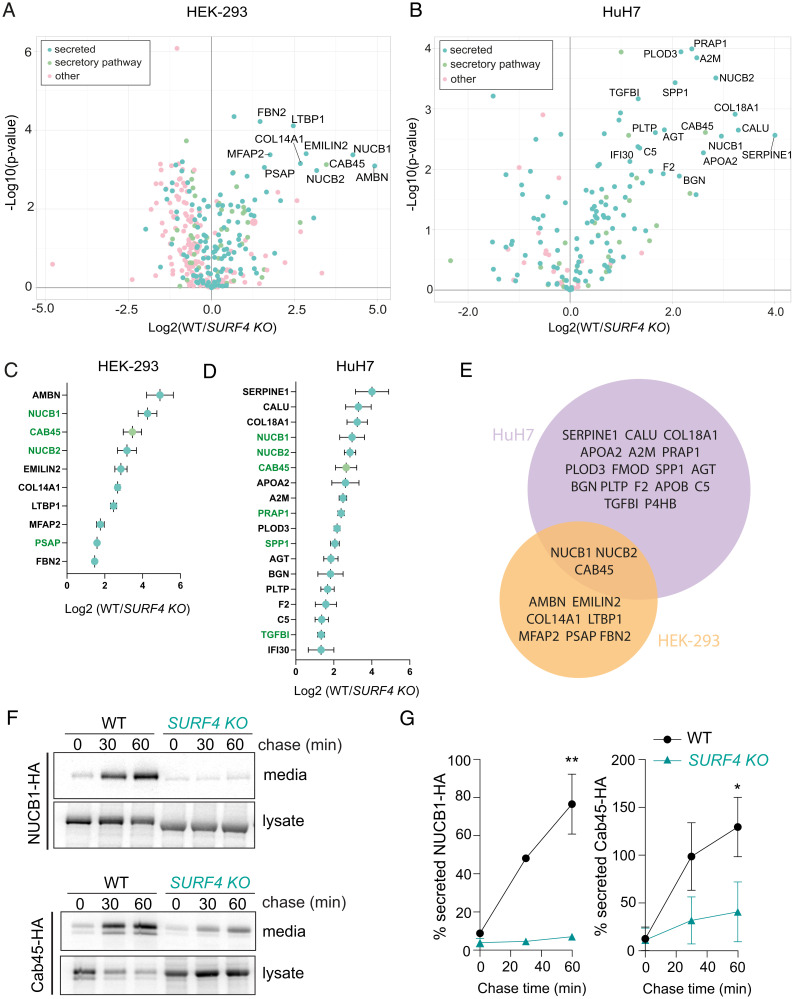
Proteomic quantification of secretion defects in SURF4 KO cells. (*A* and *B*) Volcano plot of SILAC-based quantification of proteins identified in the conditioned media of HEK-293 TREx or HuH7 WT or SURF4 KO cells. The proteins indicated with names have false discovery rate–adjusted *P* < 0.05 and log2 fold-change >1.25. (*C* and *D*) Proteins significantly depleted in the HEK-293 TREx or HuH7 SURF4 KO cell lines were ranked according to their degree of depletion. Proteins colored green contain ER-ESCAPE motifs at their predicted N termini. (*E*) Venn diagram of HEK-293 TREx and HuH7 top hits. (*F*) NUCB1-HA and Cab45-HA secretion was quantified in WT and SURF4 KO cell lines by pulse–chase with [^35^S]methionine. NUCB1 or Cab45 was immunoprecipitated with α-HA from lysates and conditioned media at the indicated times and detected by SDS-PAGE and autoradiography. Autoradiographs are representative of three independent experiments. (*G*) Quantification of NUCB1 or Cab45 secretion by phosphorimage analysis: plots show the mean ± SD of three independent experiments. Statistical test was unpaired *t* test, *P < 0.05, **P < 0.01. PAGE, polyacrylamide gel electrophoresis.

We next aimed to more broadly survey how secretion might be impacted by perturbation of the SEC24A–SURF4 interaction. We therefore sought to identify additional secretory proteins that require SURF4 to exit the ER. We used a semiquantitative proteomic approach to profile the secreted proteins in WT and SURF4 KO HEK TREx-293 and HuH7 cells. WT and SURF4 KO cells were labeled with either heavy or intermediate isotopes of arginine and lysine, including label-switch biological duplicates. After the third passage, cells were starved of methionine and then labeled with L-azidohomoalanine (AHA), a methionine analogue that enables purification of nascent proteins using click-chemistry. Conditioned media were collected, AHA-containing proteins were purified by alkyne-agarose affinity chromatography, and proteins were identified by mass spectrometry. A total of 344 and 171 proteins were identified in HEK-293 and HuH7 media fractions, respectively, with good correlation between replicate experiments (Fig. 4*A* and *B* and *SI Appendix,* Fig. S4*A* and *B*). Deletion of SURF4 resulted in significantly reduced secretion of 10 proteins in HEK-293 cells and 18 in HuH7 cells (Fig. 4*C* and *D* and Datasets S1 and S2). Consistent with previous observations, NUCB1 and NUCB2 were among the top hits affected in SURF4 KO cells (31). Nucleobindins, together with Cab45, overlap as SURF4-dependent hits between the two cell lineages (Fig. 4*E*). Many SURF4-dependent cargo proteins contain ER-ESCAPE motifs immediately downstream of the predicted signal peptide cleavage sites (*SI Appendix,* Fig. S4*C*). SERPINE1, a protein whose secretion was affected in HuH7 cells and two HEK-293 hits (FBN2 and LTBP1) do not possess ER-ESCAPE motifs but instead contain a Cardin-Weintraub (CW) motif, which has also been shown to mediate cargo–SURF4 interaction (32). Other prominent shared features of SURF4 clients include Ca binding and propensity for oligomerization (*SI Appendix,* Fig. S4*C*).

We validated two SURF4 clients identified in our proteomic analysis using pulse–chase experiments. We measured secretion of HA-tagged NUCB1 and Cab45 in WT and SURF4 KO cells. Indeed, SURF4 KO cells showed a significant reduction in release of NUCB1-HA and Cab45-HA to the media, consistent with defects in secretion (Fig. 4*C* and *D*). Secretion of NUCB1-HA could be partially rescued by transient transfection with plasmid-borne SURF4 cDNA (*SI Appendix,* Fig. S5).

### NUCB1, Cab45, and PCSK9 Use an ER-ESCAPE Motif for ER Export.

**Fig. 5.**
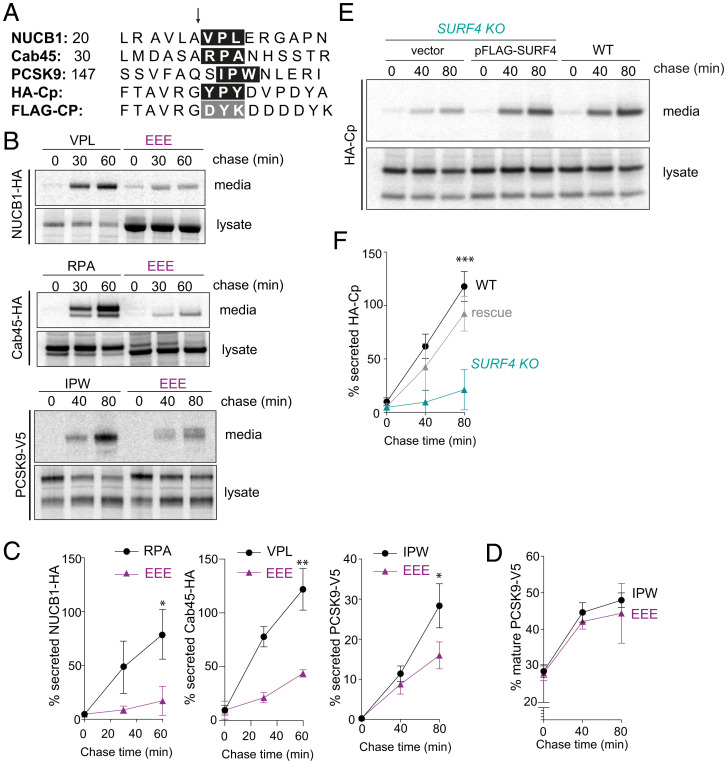
ER-ESCAPE motifs are necessary and sufficient for SURF4-mediated export. (*A*) N-terminal sequences of three bona fide SURF4 clients (NUCB1, Cab45, and PCSK9) along with a heterologous SURF4 cargo client (HA-Cp) and a bulk flow marker (FLAG-Cp) that lacks an ER-ESCAPE motif. The arrow indicates the site of signal peptide or propeptide cleavage, and the ER-ESCAPE motif is highlighted in the black box; the equivalent residues that do not form an ER-ESCAPE motif for HA-Cp are highighted in the gray box. (*B*) Secretion of NUCB1-HA, Cab45-HA, and PCSK9-V5 and their corresponding ER-ESCAPE mutants was examined by pulse–chase. (*C*) Percentage secretion was quantified by phosphorimage analysis; plots show the mean ± SD of three independent experiments. Statistical test was unpaired *t* test, *P < 0.05, **P < 0.01. (*D*) PCSK maturation was quantified by phosphorimage analysis as described in Fig. 1*B*. Plots show the mean ± SD of three independent experiments. (*E*) HA-Cp secretion was examined by pulse–chase in WT, SURF4 KO*,* and SURF4 KO cells complemented with a FLAG-SURF4 rescue plasmid. HA-Cp was immunoprecipitated with α-HA from lysates and conditioned media at the indicated times and detected by SDS-PAGE and autoradiography. (*F*) Percentage of secreted HA-Cp was quantified by phosphorimage analysis. Plots show the mean ± SD of three independent experiments. Statistical test was a one-way ANOVA with Dunnett’s correction for multiple comparisons, ***P < 0.001. PAGE, polyacrylamide gel electrophoresis.

To confirm the dependence of SURF4 clients on their ER-ESCAPE motifs (Fig. 5*A*), we mutated the ϕ-P-ϕ residues to glutamic acid, which was previously shown to impair ER export (15). Replacement of the ER-ESCAPE motifs in NUCB1 and Cab45 with an EEE peptide caused significant secretion defects (Fig. 5*B* and *C*). We note that Cab45 with the mutated ER-ESCAPE motif migrated at a lower molecular weight than the WT protein (Fig. 5*B*), suggesting impaired glycosylation associated with this mutation. To rule out indirect effects of glycosylation defects perturbing secretion, we mutated the *N*-glycosylation acceptor site in Cab45 (18), Asn40 to Gly (Cab45-N40G). This mutant migrated at a similar apparent molecular weight as Cab45-EEE but was secreted from cells relatively efficiently (*SI Appendix,* Fig. S6), confirming that glycosylation defects per se cannot explain the intracellular retention of Cab45-EEE.

PCSK9 undergoes auto-catalytic cleavage within the ER such that a propeptide region is cleaved, revealing a new N terminus. This processing is required for ER export and yields a ϕ-P-ϕ motif at the +1 position following cleavage (Fig. 5*A*). We tested whether glutamic acid substitutions in this putative ER-ESCAPE motif impaired PCSK9 secretion. Indeed, PCSK9-EEE was markedly reduced in its secretion efficiency, whereas proteolytic processing remained intact (Fig. 5*B–D*). Together, these data support the model that SURF4 clients, including PCKS9, use ER-ESCAPE motifs that are revealed either by signal peptide or propeptide cleavage.

Having demonstrated that the ER-ESCAPE motif is necessary for secretion of several SURF4 clients, we next tested whether this signal is sufficient to direct SURF4-mediated secretion. We turned again to the bulk flow marker Cp, using an HA-tagged form that exposes a YPY tripeptide after signal peptide cleavage (Fig. 5*A*). Unlike FLAG-Cp (Fig. 1*C*), which revealed DYK at the N terminus (Fig. 5*A*), HA-Cp secretion was dependent on SURF4 being reduced in the SURF4 KO line and rescued by reintroduction of SURF4 cDNA (Fig. 5*E* and *F*). These findings support the model that N-terminal ϕ-Pro-ϕ signals are necessary and sufficient to drive secretion via interaction with SURF4.

### Selective Chemical Inhibition of SURF4 Client Secretion.

**Fig. 6.**
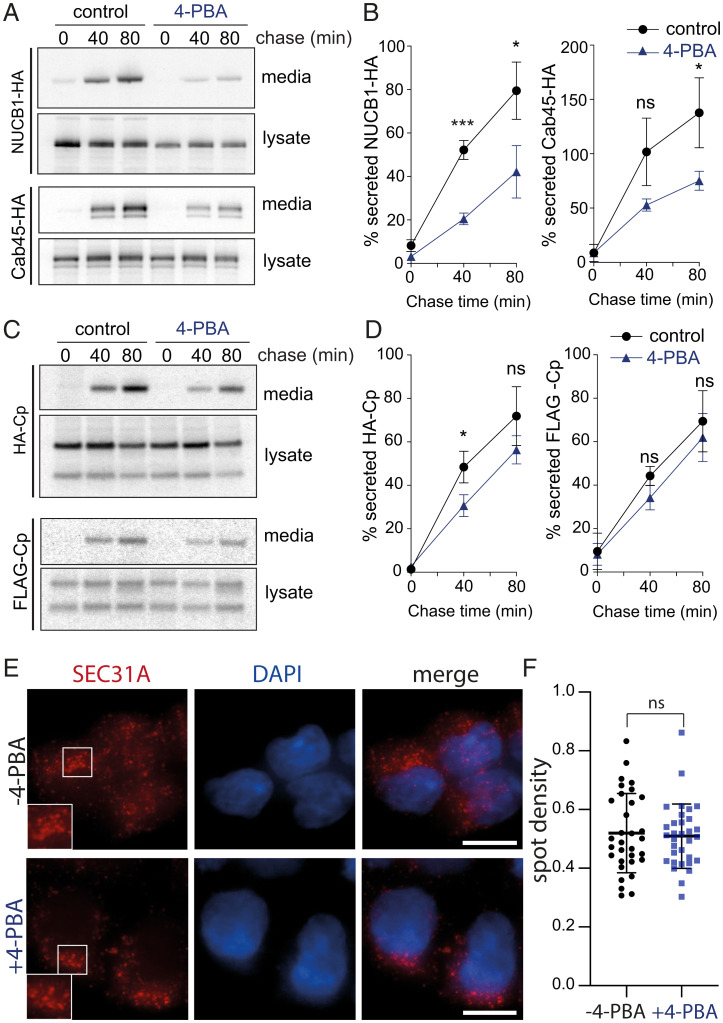
4-PBA selectively inhibits secretion of SURF4 clients but not a bona fide bulk flow marker. (*A* and *C*) Secretion of the indicated proteins was examined in the presence of 10 mM 4-PBA, which was added during the starvation phase of a pulse–chase experiment and maintained over the course of the experiment. NUCB1-HA, Cab45-HA, and HA-Cp were immunoprecipitated with α-HA from lysates and conditioned media at the indicated times and detected by SDS-PAGE and autoradiography. For FLAG-Cp, α-FLAG was used for immunoprecipitation. (*B* and *D*) Percentage secretion into the media was quantified by phosphorimage analysis. Plots show the mean ± SD of three independent experiments. Statistical test was an unpaired *t* test, *P < 0.05, ***P < 0.001. (*E*) HEK-293T cells were left untreated or incubated with 10 mM 4-PBA for 4 h and then fixed, immunostained for Sec31A, stained with DAPI, and subjected to fluorescence microscopy. Insets in the left panels show higher magnification of the boxed areas. Scale bars, 10 µm. (*F*) Quantification of the density of SEC31A-postivie puncta in the presence and absence of 4-PBA. Values represent the number of SEC31A-positive puncta identified per cell. A total of 32 cells from each condition were measured. Error bars represent SD; statistical test was an unpaired *t* test. ns, not significant; PAGE, polyacrylamide gel electrophoresis.

We next tested whether SURF4 clients were differentially impaired in their secretion by 4-PBA–mediated occlusion of the SEC24 B-site. Similar to the effects observed for PCSK9, secretion of NUCB1-HA and Cab45-HA was reduced in the presence of 4-PBA (Fig. 6*A*). In addition, secretion of the heterologous protein HA-Cp, which carries an ER-ESCAPE motif, was also reduced by the presence of the small molecule, albeit to a lesser extent than PCKS9, NUCB1, or Cab45 (Fig. 6*C*). In contrast, the bona fide bulk flow marker FLAG-Cp, which lacks the SURF4 export signal, was unaffected (Fig. 6*C* and *D*).

Despite the lack of effect on bulk secretion, we were concerned that 4-PBA might perturb secretion more broadly. Protein export from the ER generally occurs via discrete ERESs, which are sites of cargo concentration and vesicle formation. To further explore the possibility of a broader effect of 4-PBA on protein export, we examined the localization of the COPII coat protein, SEC31A, which marks ERESs in cells (19), in the presence of 4-PBA. Using immunofluorescence microscopy, we observed that when HEK293 cells were treated with 10 mM 4-PBA for 4 h prior to fixation and immunolabeling for SEC31A, the distribution and number of ERESs was unaffected (Fig. 6*E* and *F*). These results suggest that the action of 4-PBA is specifically linked to its occlusion of the SEC24 B-site. In order to investigate the broader effects of 4-PBA in secretion, we performed secretome analysis using SILAC and AHA labeling. After passage in media containing either light or heavy arginine and lysine isotopes, WT HEK-293 cells were labeled with AHA or AHA plus 10 mM 4-PBA for 4 h. Media were collected, and secreted proteins were recovered and quantified as described above. Secretion of relatively few proteins was reduced upon treatment with 4-PBA, with SURF4 clients the most prominent proteins affected (*SI Appendix,* Fig. S7*A* and *B* and Datasets S3). Most secretory proteins detected were not affected by 4-PBA treatment (*SI Appendix,* Fig. S7*C*).

## Discussion

Selective ER export relies on a series of protein–protein interactions that specify loading of secretory proteins into nascent transport vesicles. At the top of this interaction pyramid is Sec24, the adaptor protein of the COPII coat (3). All organisms have multiple Sec24 isoforms, and each isoform likely uses multiple sites of interaction to drive binding of diverse secretory cargo and cargo receptors. This hierarchical arrangement ensures capture of the full spectrum of protein sequence and structure that uses this pathway (6, 11, 12, 20, 21). In yeast, four different cargo-binding sites, referred to as A-, B-, C-, and D-sites, on Sec24 allow recognition of a diverse set of sorting signals (7, 8, 22). Proteomics of vesicles generated with different mammalian Sec24 isoforms revealed both shared and isoform-specific clients (11). The nature of specialized cargo-binding events for distinct Sec24-binding sites raises the appealing possibility of selective inhibition of secretion, an otherwise essential process. Here, we have mapped the interaction network responsible for secretion of the cholesterol regulator PCSK9. We confirmed that SEC24A and the cargo receptor SURF4 drive PCSK9 secretion and demonstrated a role for the B-site of SEC24A in this process. A known small molecule, 4-PBA, which binds the SEC24 B-site (16), inhibits the SEC24A–SURF4 interaction and impairs secretion of PCSK9 and other SURF4 clients. This establishes the principle that small-molecule modulation of cargo selection can selectively alter the cellular secretome.

4-PBA was first described as a chemical chaperone thought to improve ER folding capacity (23). Generally, 4-PBA suppresses cellular responses against the accumulation of misfolded proteins in the ER, such as the unfolded protein response (UPR), in both yeast and mammalian cells (24–27); however, the mechanism of action of 4-PBA was unknown. Recently it has been described that 4-PBA occludes cargo recognition sites in the COPII coat, generating cargo-packaging defects that result in increased capture of ER resident proteins (16). That observation explains the previously described effects of 4-PBA facilitating trafficking of misfolded proteins (28, 29). 4-PBA mimics the structure of C-terminal hydrophobic sorting signals that interact with the SEC24 B-site (16). This relatively broad-spectrum effect of 4-PBA is consistent with the conserved nature of the Sec24 B-site across all SEC24 isoforms and highlights its limited utility as a selective therapeutic. However, structural studies have shown that subtle changes in the vicinity of the B-site of human SEC24C and SEC24D results in a marked specificity for distinct export signals (6), raising the prospect that more refined small molecules might be able to selectively target different B-site isoforms. Indeed, understanding the molecular basis for SEC24A discrimination of SURF4 is an important question that should inform additional chemical approaches.

In addition to the SEC24–SURF4 interaction, secretion of PCSK9 and other SURF4-dependent cargoes relies on interaction between an unknown SURF4 luminal domain and the ER-ESCAPE motifs of its clients (15). This interaction represents another potentially druggable interface that might be similarly targeted by small-molecule inhibition. Since multiple diverse cargo proteins interact with the Sec24 B-site (6–8, 16), perturbing SURF4–client interaction may represent a more tractable way forward to therapeutic intervention. However, the client repertoire of SURF4 seems substantial (15, 30, 31), and being able to selectively inhibit a subset of receptor–cargo interactions may be difficult. Nonetheless, like SEC24, SURF4 also appears to be a multivalent export facilitator, since it also recognizes a second ER export signal, the CW motif, found on Shh (32). Discerning which SURF4 clients use the ER-ESCAPE versus CW motifs and defining the sites on SURF4 that mediate these interactions are other important open questions that should inform future small molecule–screening approaches. We also note that multiple SURF4 clients are Ca-binding proteins and/or undergo oligomerization, consistent with previous observations that suggested that SURF4 is important to (i) prevent premature oligomerization within the ER and (ii) rapidly remove these proteins from the Ca-rich ER lumen before inappropriate oligomerization (15). Whether a putative chaperone-like function is also mediated by the ER-ESCAPE (or CW) motif or is instead conferred by a separate domain of SURF4 remains to be determined, although no obvious induction of the UPR has been observed upon the loss of SURF4 (30–33). Dissecting cargo-binding and chaperone functions of SURF4 and understanding how interactions at these sites on SURF4 inform the interaction with SEC24 will be an important priority.

Our findings specifically raise the prospect of selective inhibition of PCSK9 secretion. In recent years, PCSK9 has emerged as a key therapeutic target for lowering circulating low-density lipoprotein (LDL) levels. Monoclonal antibodies directed against PCKS9 not only markedly lower LDL levels but also prevent cardiovascular events. However, PCSK9 monoclonal therapeutics require monthly injections, and their manufacturing process is expensive. Therefore, non-antibody treatments to inhibit PCSK9 function are actively being developed as alternative therapies (34, 35). Our work here establishes that small-molecule inhibition of the secretory pathway may be a useful alternative to inhibit PCSK9 function. Indeed, 4-PBA has been shown to reduce atherosclerosis in mice (36, 37), although the molecular basis for this effect is not known. Finally, given the wealth of structural information on cargo selection by SEC24, additional small-molecule screening based on the types of protein–protein interaction assays described here suggests a path forward for more specific inhibition of protein secretion and raises the possibility for systemic modulation of other health-related secretory proteins.

## Materials and Methods

### Cell Lines.

HEK TREx-293 and HuH7 cell lines used in this study were cultured in Dulbecco’s Modified Eagle’s Medium (DMEM; Gibco) with 10% fetal bovine serum (Qualified FBS, Gibco). HEK TREx-293 SURF4 KO was previously described (31). For CRISPR-Cas9–mediated KO of Sec24A and Sec24B, guide RNAs targeting exon 1 (5′-GGCCCAGAACGGAGCCGCCT-3′) of SEC24A and exon 3 of SEC24B (5′-CGTATCCTAGTGTTTCATAT-3′) were designed using the CRISPR design tool (https://benchling.com) and cloned into pX458 (pSpCas9 BB-2A-GFP; Addgene, plasmid #48138). Multiple single cell–derived KO clones per each guide RNA were isolated and screened for gene disruption by Western blotting. SEC24C and SEC24D KO cell lines were described previously (38). Cells were validated by immunoblotting or quantitative RT-PCR (*SI Appendix,* Fig. S8). Transient transfections were performed with TransIt 293 reagent from Mirus. All cell lines were routinely checked for mycoplasma contamination and tested negative.

### Constructs and Reagents.

The plasmids used in this study are listed in *SI Appendix,* Table S1. PCSK9 for mammalian expression in the pcDNA5 vector containing a C-terminal V5 tag was generated by GeneScript. PCSK9-EEE mutant contained three-point mutations within the ER-ESCAPE motif, I154E, P155E, and W156E, and was generated using the QuikChange Lightning Multi Site-Directed Mutagenesis Kit (Agilent Technologies). NUCB1 for mammalian expression in the pcDNA3.1 vector containing a C-terminal HA tag was generated by GeneScript. The NUCB1-EEE mutant contained three-point mutations within the ER-ESCAPE motif, V27E, P28E, and L29E, and was generated using the QuikChange Lightning Multi Site-Directed Mutagenesis Kit (Agilent Technologies). The Cab45 plasmid for mammalian expression in the pLPCX vector containing a C-terminal HA tag was kindly provided by Julia Von Blume (Yale School of Medicine, New Haven, CT, USA) (39). The Cab45-EEE mutant contained three-point mutations within the ER-ESCAPE motif, R37E, P38E, and A39E, and was generated using the QuikChange Lightning Multi Site-Directed Mutagenesis Kit (Agilent Technologies). SURF4 for mammalian expression in the pcDNA3.1 vector containing an N-terminal FLAG tag was generated by GeneScript. The HA-Cp construct in the pSV‐SPORT1 (4) was kindly provided by Ari Helenius (Institute of Biochemistry, ETH Zurich, Switzerland). The FLAG-tagged version of Cp was created by sequential mutagenesis reactions using the QuikChange Lightning Multi Site-Directed Mutagenesis Kit (Agilent Technologies).

HaloTag, SEC24A, SEC24C, and SURF4 plasmids for the NanoBiT protein-protein interaction (PPI) assays were in the pcDNA3.1 vector containing the SmBiT tag for HaloTag, SEC24A and SEC24C, and the LgBiT tag for SURF4. The SEC24A open reading frame (ORF) was amplified by PCR from a commercially available plasmid [cDNA SEC24A in pcDNA3.1^+/−^C-(K)-DYK, GeneScript] and subcloned into the pFC36K-SmBiT and pFN35k-SmBiT backbones including the TK promoter (Promega). SURF4 ORF was amplified by PCR from a commercially available plasmid [cDNA SURF4 in pcDNA3.1^+/−^C-(K)-DYK, GeneScript] and subcloned into the pFC34-LgBiT and pFN33-LgBiT backbones including the TK promoter (Promega). These constructs resulted in very low expression of SEC24A and SURF4 under the TK promoter. The fragments containing SmBiT-SEC24A or SEC24A-SmBiT and LgBiT-SURF4 or SURF4-LgBiT were PCR amplified and subcloned into the HindIII/NotI sites of pcDNA3.1. The HaloTag-SmBiT fragment was PCR amplified from the commercially available plasmid *(*Promega, Catalog No. N202A) and also subcloned into pcDNA3.1. The SEC24A B- and C-site mutants were generated on pcDNA3.1-SmBiT-SEC24A using QuikChange Multi Site-Directed Mutagenesis (Agilent Technologies).

4‐PBA (Sigma-Aldrich) and 5‐PVA (Sigma-Aldrich) were dissolved in an equimolar amount of NaOH to prepare a 0.5-M stock solution with pH 7.

### Pulse–Chase.

Cells were starved in Methionine/Cysteine-free DMEM (Gibco, #21013024) for 30 min, pulsed with 15 uCi/mL ^35^Smethionine/cysteine (EasyTag EXPRESS ^35^S, PerkinElmer) for 30 min and chased in complete DMEM containing 10% FBS. At each time point, media and cells were collected and cells were lysed in Buffer A (50 mM Tris, pH 7, 150 mM NaCl, 1% [vol/vol] Triton X-100, and 2 mM ethylenediaminetetracetic acid (EDTA)) supplemented with Protease inhibitors (Complete EDTA-free, Roche). Media and cell lysates were precleared, and the protein of interest was immunoprecipitated. Radiolabeled immunoprecipitated proteins were eluted in Loading buffer (50 mM Tris, pH 6.8, 0.1% [vol/vol] glycerol, 20% [p/v] SDS, 5% [vol/vol] b-mercaptoethanol, and 1 mg/mL Bromophenol Blue), separated on NuPAGE 4 to 12% Bis-Tris gels (Thermo Fisher Scientific), and detected by phosphorimaging using a Typhoon scanner (GE Healthcare). The protein bands were quantified using Fiji, and the percentage of the mature or secreted band in each sample was plotted with Prism 7.0 (GraphPad Software). For experiments with 4-PBA, compound was added to a final concentration of 10 mM during the starvation phase and included at the same concentration in the pulse and chase media.

### SILAC-AHA Labeling and Enrichment of Endogenously Secreted Proteins.

In order to elucidate SURF4-dependent secretome, the method described in ref. (40) was adapted. To ensure SILAC label incorporation, WT and *SURF4 KO* TREx-293 cells were grown for three passages in DMEM (AthenaES, #0420) supplemented with 10% FBS and with either heavy (84 µg/mL L-arginine-HCl, 13C6, 15N4, Thermo Fisher Scientific, #89990; 145 µg/mL L-lysine 2HCl, 13C6, 15N2, Thermo Fisher Scientific, #88209) or intermediate (84 µg/mL L-arginine-HCl 13C6, Thermo Fisher Scientific, #88210; 146 µg/mL L-lysine-2HCl, 4,4,5,5,D4, Thermo Fisher Scientific, #88437) SILAC labels, supplied with excess L-proline (200 µg/mL, Formedium LTD, #DOC0177) and L-methionine (201 µM, AthenaES, #0419). Cells at 70% confluency in 10-cm dishes were starved in SILAC DMEM with no methionine for 30 min and then labeled with AHA (0.5 mM; AnaSpec, #AS-63669) for 20 h. Oppositely labeled media were then combined and treated with 0.5 M aminoguanidine-HCl (Sigma-Aldrich, #396494) and protease inhibitors and centrifuged to remove remaining cells. Samples concentrated to ∼250 μL (Merck, Amicon, 3-kDa cutoff) were combined with 250 μL lysis buffer (1% 3-[(3-cholamidopropyl)dimethylammonio]-1-propanesulfonate [CHAPS], 50 mM 4-(2-hydroxyethyl)-1-piperazineethanesulfonic acid (Hepes), pH 7, 150 mM NaCl, and 8 M urea) with protease inhibitors (PIs). Alkyne agarose slurry (100 μL; Jena Bioscience, #CLK-1032-2) was washed with immunoprecipitation (IP) buffer (1% CHAPS, 50 mM Hepes, pH 7, and 150 mM NaCl). The washed resin, 2 mM CuSO_4_ (Chem Cruz, #sc-203009A), 2 mM tris-hydroxypropyltriazolylmethylamine (THPTA) (Jena Bioscience, #CLK-1010-25), and 4 mM sodium ascorbate (Sigma-Aldrich, #PHR1279) were added to the samples, and these were rotated for 16 to 20 h at room temperature. The resin was then washed with high-performance liquid chromatography (HPLC)–grade water (Thermo Fisher Scientific, #W/016/17), resuspended in sodium dodecyl sulfate (SDS) buffer (100 mM Tris⋅HCl, pH 8, 1% SDS, 250 mM NaCl, and 5 mM EDTA) with 10 mM dithiothreitol (DTT) and incubated at 70° for 15 min. Once cooled, samples were incubated in the dark for 30 min with SDS buffer (100 mM Tris–HCl, pH 8, 1% SDS, 250 mM NaCl, and 5 mM EDTA) with 40 mM iodoacetamide (Sigma-Aldrich, #I1149). The resin was then resuspended in SDS buffer, transferred to a spin column, and washed with SDS buffer, 8 M urea in 100 mM Tris, pH 8, and 20% acetonitrile (MeCN). The resin was finally resuspended in digestion buffer (100 mM Tris⋅HCl, pH 8, 2 mM CaCl_2_, and 10% MeCN) and processed for mass spectrometry analysis.

### Mass Spectrometry and Data Analysis.

Proteins retained in the resin were digested with trypsin (Promega) overnight at 37 °C. After digestion, samples were centrifuged and each supernatant was transferred to a fresh tube. Then, beads were extracted with 50% MeCN/0.5% formic acid (FA) and combined with the corresponding supernatant. Peptide mixtures were phase-reverse fractionated, partially dried in Speed Vac, and desalted using a homemade C18 (3M Empore) stage tip that contained 3 μL porous R3 (Applied Biosystems) resin. Bound peptides were eluted sequentially with 30%, 50%, and 80% MeCN in 0.5% FA and concentrated in a Speed Vac (Savant). The peptide mixtures were desalted and fractionated using self-packed C18 (3M Empore) stage tips filled with 3 μL porous R3 resin (Applied Biosystems). The stage tips were activated and equilibrated with 50% MeCN, 80% MeCN/0.5% FA. Peptide mixtures were loaded onto stage tips and washed with 0.5% FA. Then, stage tips were equilibrated with 100 μL of 10 mM ammonium bicarbonate for pH = 8 fractionation. Bound peptides were sequentially eluted using 8 × 20 μL of 10 mM ammonium bicarbonate containing increasing concentrations of MeCN and combined into four fractions. Eluted peptides were acidified and concentrated in a Savant Speed Vac.

Peptides were then subjected to liquid chromatography/mass spectrometry/mass spectrometry (LC/MS2) analysis. Desalted peptide mixtures were separated using an Ultimate 3000 RSLC nano System *(*Thermo Fisher Scientific*)* with an MeCN gradient, consisting of buffer A (2% MeCN, 0.1% FA) and buffer B (80% MeCN, 0.1% FA) at a flow rate of 300 nL/min for 2 h. The HPLC system was coupled to a Q-Exactive Plus hybrid quadrupole-Orbitrap mass spectrometer (Thermo Fisher Scientific) equipped with a nanospray ion source. The mass spectrometer was operated in standard data-dependent mode, performed survey full-scan (mass spectrometry (MS), *m/z* = 380 to 1,600) with a resolution of 70,000, followed by mass spectrometry/mass spectrometry (MS2) acquisitions of the 15 most intense ions with a resolution of 17,500 and normalized collision energy (NCE) of 27%. MS target values of 1e6 and MS2 target values of 1e5 were used. The isolation window was set as 1.5 *m/z* and dynamic exclusion for 30 s.

The acquired MS2 raw files were processed using MaxQuant with the integrated Andromeda search engine (v1.6.6.0). MS2 spectra were searched against the Homo sapiens Reviewed, UniProt Fasta database (March 2019). Carbamidomethylation of cysteines was set as fixed modification, while oxidation of methionine, N-terminal protein acetylation, Met replaced by AHA, and reduction of Met replaced by AHA as variable modifications. For AHA-SILAC, Lys0/Arg0 (light), Lys4/Arg6 (intermediate), and Lys8/Arg10 (heavy) were specified as metabolic labels. Enzyme specificity was set to trypsin/p, and maximum two missed cleavages were allowed. Protein quantification requires one (unique+razor) peptide. Further data visualization and analysis were carried out using Excel, Perseus (v1.6.2.3), and R (v3.6.1). A protein hit was subjected for further analysis if it had at least two SILAC ratios in four replicates and two or more detected peptides. One-sample *t* test was used to calculate *P* values, which were then adjusted for multiple testing with the Benjamini-Hochberg method.

### Cell Proliferation Assays: MTS Assays.

Cells were seeded into 96-well plates at a density of 8 × 10^3^ cells/well (100 μL) and treated the following day with 5, 10, 20 mM 4-PBA or 5, 10, 20 mM 5-PVA for 4 h. For the MTS assay, MTS Cell Proliferation Colorimetric Assay Kit (Generon) was used following the manufacturer’s instructions. Briefly, 2 h before the desired time point, 10 µL of the MTS reagent was added into each well, and cells were incubated at 37 °C for 2 h. The absorbance was detected at 490 nm with a Microplate Reader (Spark plate reader, Tecan).

### Western Blot Analysis.

For analysis of total cellular proteins, cells were lysed in 100 mM Tris, pH 8.0, with 1% SDS supplemented with protease inhibitor (Complete EDTA-free, Roche). Cells in lysis buffer were heated three times for 5 min at 95 °C. Protein concentrations were adjusted based on A_280_ values to a final concentration of 2.5 μg/μL, and loading buffer was added; 25 μg of proteins was then separated in NuPAGE 4 to 12% Bis-Tris gels (Thermo Fisher Scientific), transferred to 0.2-μm nitrocellulose membrane (Whatman), and detected with the corresponding antibody. Chemiluminescence was visualized employing horseradish peroxidase (HRP)–conjugated secondary antibodies and chemiluminescent substrate (Immobilon Western Chemiluminescent HRP Substrate, Sigma-Aldrich).

### Immunofluorescence Microscopy.

HEK-293 TREx cells were seeded on coverslips in 6-well plates and then treated with or without 10 mM 4-PBA for 4 h. Then, cells were fixed with 4% paraformaldehyde for 15 min, permeabilized with 0.1% Saponin for 10 min, blocked with 1% bovine serum albumin for 1 h, and stained with 1:100 anti-SEC31A (BD Biosciences, #612350) overnight at 4 °C. The next day, coverslips were stained with anti-mouse 1:250 Alexa Fluor 568 (Thermo Fisher Scientific, #A11031) for 1 h at room temperature. Coverslips were washed 3 × 3-min with phosphate-buffered saline after fixing, primary and secondary antibody incubations; cells were stained with 10 μg/μL DAPI (Merck) for 15 min. Coverslips were mounted in Prolong Diamond Antifade (Thermo Fisher Scientific). Z-stack images at 0.9-μm intervals were taken on a Nikon Ti2 Eclipse microscope with a 60x/1.4 numerical aperture oil immersion objective and an Orca-flash 4.0 camera (Hamatsu). Images were collected using NIS Elements AR 5.11.03 software, followed by processing in Fiji. SEC31A puncta were quantified from merged Z-stack images using an in-house Fiji macro (41).

### PPI Assays.

For PPI luminescence measurements, double KO (*SEC24AKO SURF4KO*) TREx-293 cells were plated at a density of 8 × 10^3^ cells/well (100 μL) in DMEM and transfected the following day with the correspondent NanoBiT constructs. Twenty-four hours after transfection, growth medium was exchanged with Opti-MEM, and cells were incubated for 3.5 h at 37 °C. Nano-Glo Live Cell Substrate (Promega) was added, and luminescence was measured using a Spark plate Reader (Tecan) at 37 °C.

## Supplementary Material

Supplementary File

Supplementary File

Supplementary File

Supplementary File

## Data Availability

All study data are included in the article and/or supporting information.
