# Supplementary material for: Selective inhibition of protein secretion by abrogating receptor–coat interactions during ER export
Source: Proc Natl Acad Sci U S A. 2022 Jul 28;119(31):e2202080119. doi: 10.1073/pnas.2202080119 (PMC9351455; doi:10.1073/pnas.2202080119)
Supplement: Supplementary File [file pnas.2202080119.sapp.pdf]

PNAS

Supplementary Information for

Selective inhibition of protein secretion by abrogating receptor-coat interactions during ER export.

Natalia Gomez-Navarro, Julija Maldutyte, Kristina Poljak, Sew-Yeu Peak-Chew, Jonathon Orme, Brittany J. Bisnett, Caitlin H. Lamb, Michael Boyce, Davide Gianni, Elizabeth A. Miller

Elizabeth A. Miller
Email: emiller@mrc-lmb.cam.ac.uk

This PDF file includes:

Figures S1 to S8
Table S1

Other supplementary materials for this manuscript include the following:

Datasets S1 to S3

Figure S1

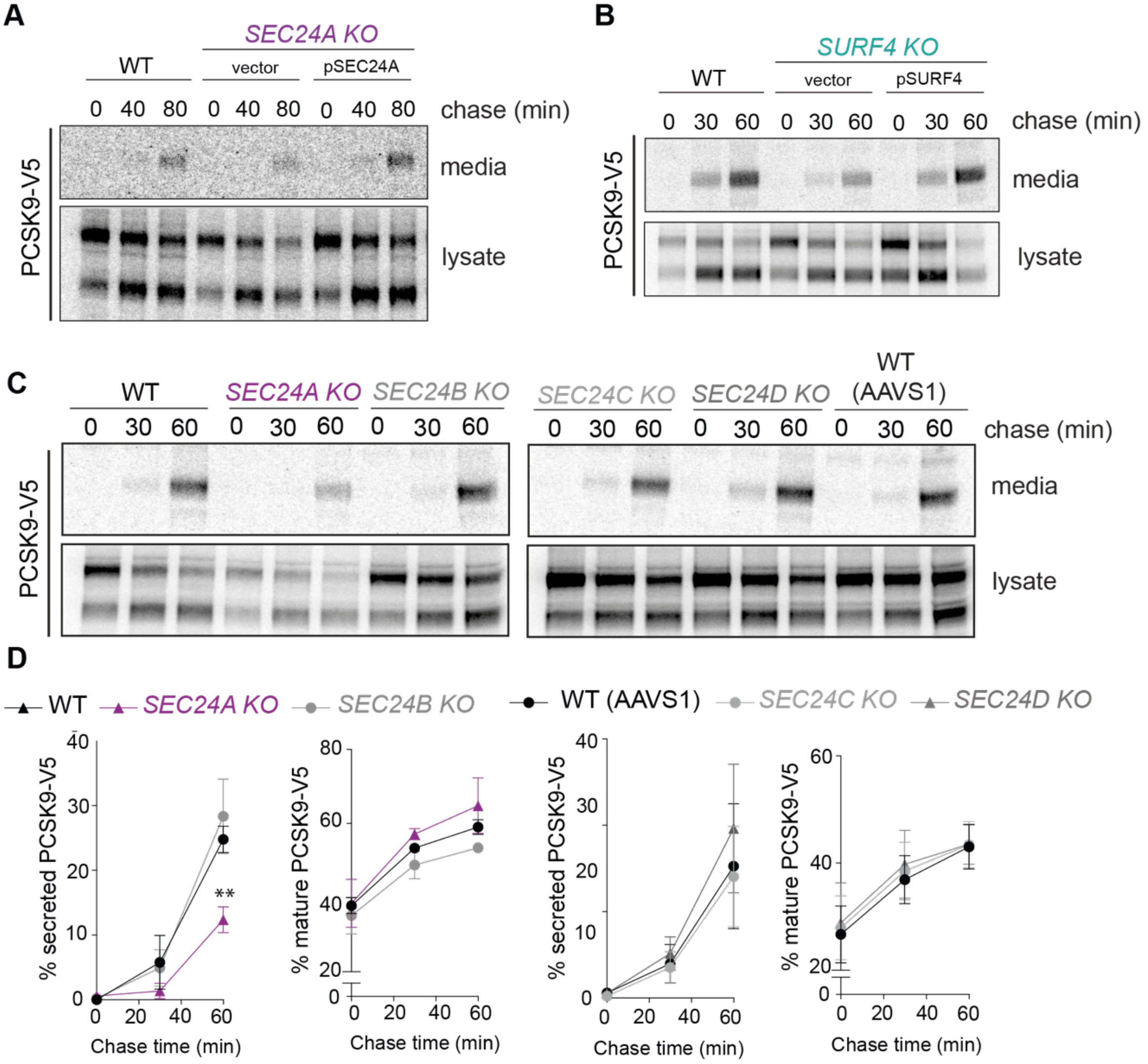

Figure S1: Rescue of PCSK9 secretion defects in SEC24A KO and SURF4 KO cells. (A) PCSK9-V5 secretion was examined by pulse-chase in WT, and *KO* cells complemented by transient transfection with the relevant rescue plasmids. PCSK9 was immunoprecipitated with α-V5 from lysates and the conditioned media at the indicated times and detected by SDS-PAGE and autoradiography as described in Figure 1. **(B)** Quantification of PCSK9 secretion shown in A as described in Figure 1. **(C)** PCSK9-V5 secretion was monitored in *SEC24* KO lines indicated, along with the corresponding wild-type lines (HEK TREx293 for *SEC24B* KO and AAVS1 for *SEC24C* and *SEC24D* KO), as described in Figure 1. **(D)** Quantification of PCSK9 secretion shown in C as described in Figure 1.

Figure S2

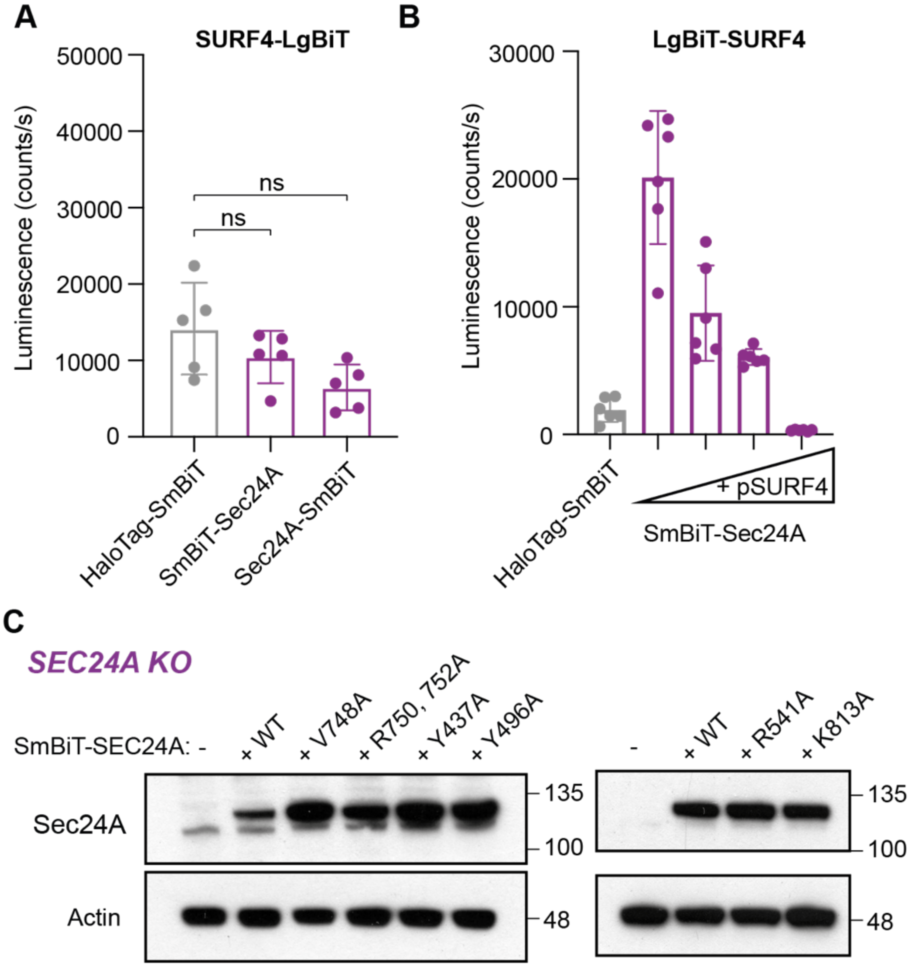

Figure S2: Establishment of the SEC24A-SURF4 NanoBiT assay and stability of SEC24A mutants. (A) Control experiments for the NanoBiT assay showing non-optimal tagging orientations and the corresponding negative control in *SEC24A SURF4* double KO cells. The graph shows mean luminescence ± SD (n = 6); statistical test was a one-way ANOVA with Dunnett's correction for multiple comparisons. **(B)** Specificity of the assay was tested by expressing increasing amounts of untagged fusion partners. Plots show mean ± SD, n=6. **(C)** Stability of B-site (left-hand panel) and C-site (right-hand panel) SEC24A mutants was analysed by immunoblotting cell lysates prepared from equal numbers of cells with α-SEC24A antibodies.

Figure S3

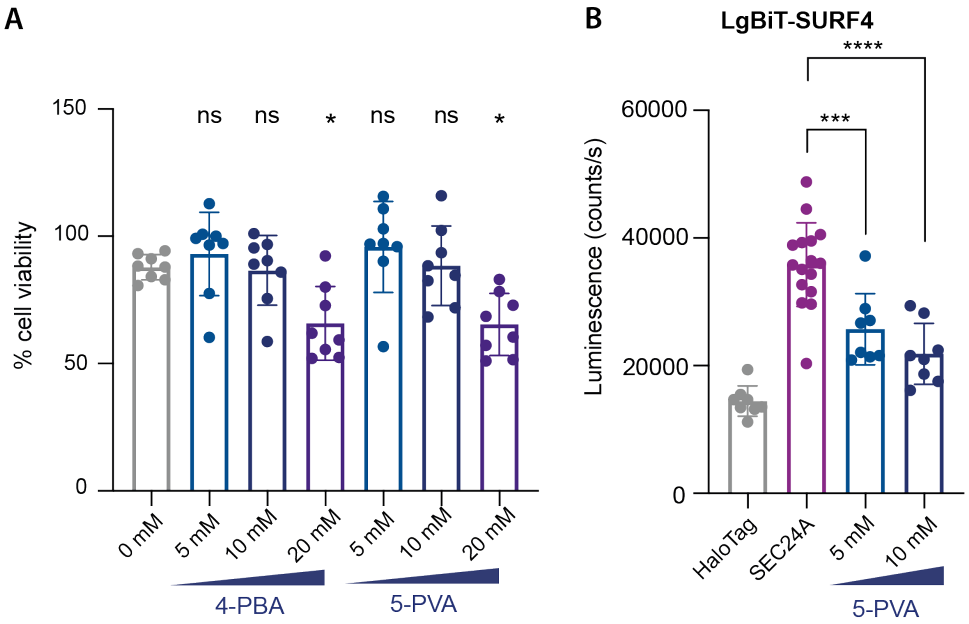

Figure S3: Effects of 4-PBA on cell viability. (A) Dose-dependent effects of 4-PBA on cell viability. Cells were grown on plates overnight and then treated for 4h with the indicated concentrations of 4-PBA. Cell viability was measured using the MTS Cell Proliferation Colorimetric Assay Kit. Values are given as mean ± SD, n=8. **(B)** Luminescence that reports on the interaction between SURF4 and SEC24A was measured in the presence of the indicated concentrations of 5-PVA. The NanoBiT reporters were induced overnight and cells were treated with 5-PVA for 4h. The graph shows the mean luminescence ± SD (n = 8; n= 16 for 0 mM 4-PBA); statistical test was a one-way ANOVA with Dunnett's correction for multiple comparisons, * p<0.05, **** p<0.0001.

Figure S4

A

HEK-293

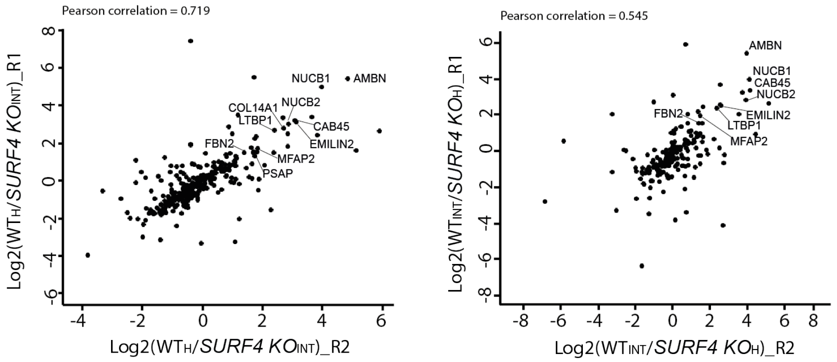

B

HuH7

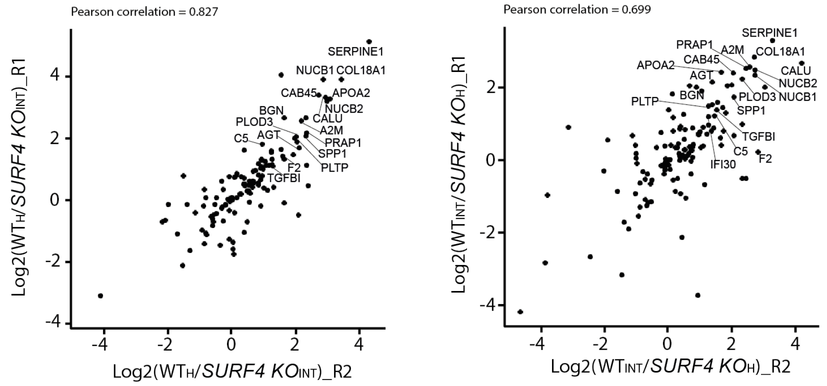

C

	Protein:	N-terminus:	CW motif?	Ca-binding?	oligomer?
Shared	NUCB1	Val-Pro-Leu	-	+	+
	CAB45	Arg-Pro-Ala	-	+	+
	NUCB2	Val-Pro-Ile	-	+	+
	CALU	Lys-Pro-Thr	-	+	-
	COL18A1	Asn-Leu-Leu	-	-	+
HEK-293	AMBN	Val-Pro-Phe	-	-	+
	SERPINI1	Thr-Gly-Ala	-	-	-
	EMILIN2	Gly-Pro-Gln	-	-	+
	COL14A1	Gln-Val-Ala	-	-	+
	LTBP1	Arg-Leu-Arg	+	+	+
	NBL1	Ala-Pro-Pro	-	-	-
	GAS6	Leu-Pro-Ala	-	+	+
	MFAP2	Gln-Gly-Gln	-	-	+
	PSAP	Gly-Pro-Val	-	-	-
	FBN2	Gln-Pro-Gln*	+	+	+
HuH7	SERPINE1	Val-His-His	+	-	-
	APOA2	Ala-Leu-Val	-	-	+
	A2M	Ser-Val-Ser	-	-	+
	PRAP1	Val-Pro-Val	-	-	-
	PLOD3	Ser-Asp-Arg	-	-	-
	FMOD	Gln-Tyr-Glu	-	-	?
	SPP1	Ile-Pro-Val	-	-	+
	AGT	Asp-Arg-Val	-	-	-
	BGN	Leu-Pro-Phe	-	-	+
	PLTP	Glu-Phe-Pro	-	-	-
	F2	Gln-His-Val	-	+	-
	APOB	Glu-Glu-Glu	-	-	-
	C5	Asp-Arg-Val	-	-	-
	TGFBI	Gly-Pro-Ala	-	-	+
	P4HB	Asp-Ala-Pro	-	-	-

Figure S4: ER-ESCAPE motif sequence of the top SURF4 cargo clients identified in the proteomic quantification. (A), (B) Scatter plots of Log2 SILAC ratios of the two replicates within each label-switch pair in HEK-293 or HuH7 cell media samples. Correlation is indicated by Pearson's correlation coefficient. **(C)** Top SURF4 client hits identified by proteomic analysis and annotated for (i) N-terminal sequence color coded based on relative contribution of each amino acid position to the strength of the ER-ESCAPE motif as described in (Yin et al., 2018) (green = very good, yellow = good, black = neutral, and red = bad); (ii) presence of CW motif; (iii) presence of a Ca-binding domain or annotation as a Ca-binding protein; (iv) propensity to oligomerize.

Figure S5

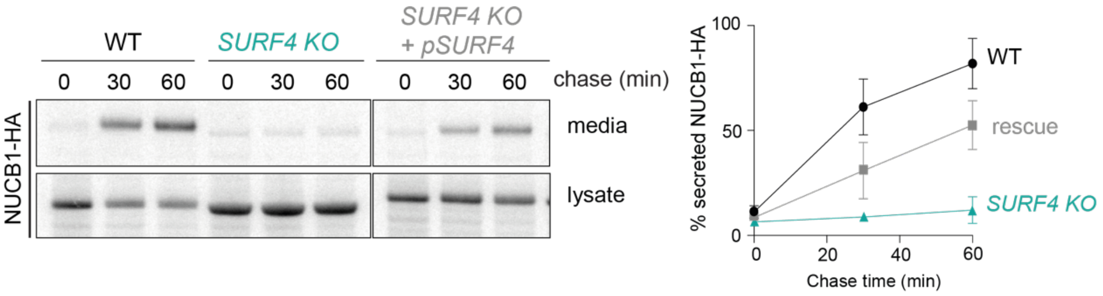

Figure S5: Rescue of NUCB1 secretion defects in SURF4 KO cells. NUCB1-HA secretion was examined by pulse-chase in WT, *SURF4 KO,* and *SURF4 KO* cells complemented with the pFLAG-SURF4 plasmid. NUCB1-HA was immunoprecipitated with α-HA antibodies from lysates and the conditioned media at the indicated times and detected by SDS-PAGE and autoradiography.

Figure S6

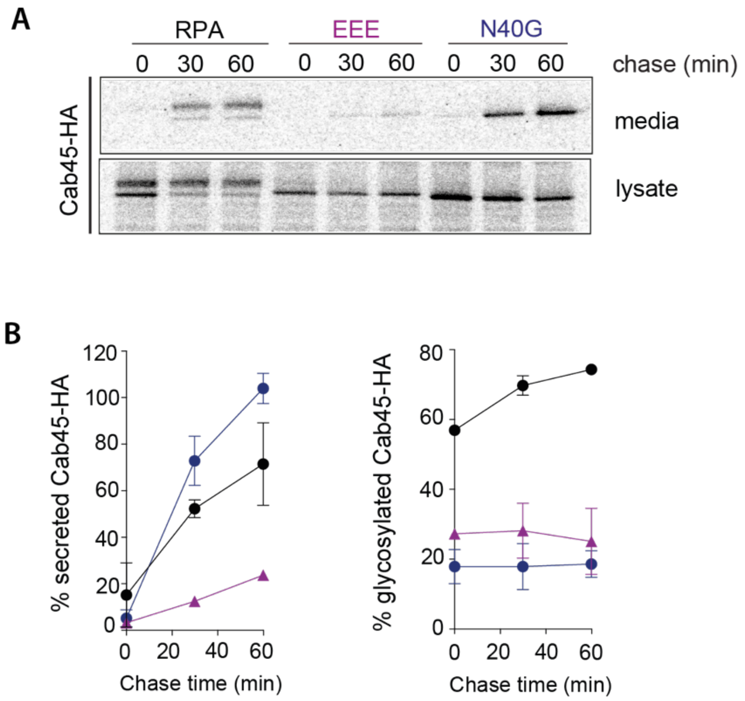

Figure S6: Glycosylation defects do not influence Cab45 secretion. A) Cab45-HA secretion was examined by pulse-chase in cells expressing WT (RPA), ER-ESCAPE mutant (EEE) and glycosylation mutant (N40G) variants. Cab45-HA was immunoprecipitated with α-HA antibodies from lysates and the conditioned media at the indicated times and detected by SDS-PAGE and autoradiography. **(B)** Percentage secretion into the media was quantified by phosphorimage analysis. Percentage of glycosylation was quantified as [glycosylated signal / [total protein (glycosylated + unglycosylated). Plots show the mean ± SD of three independent experiments.

Figure S7

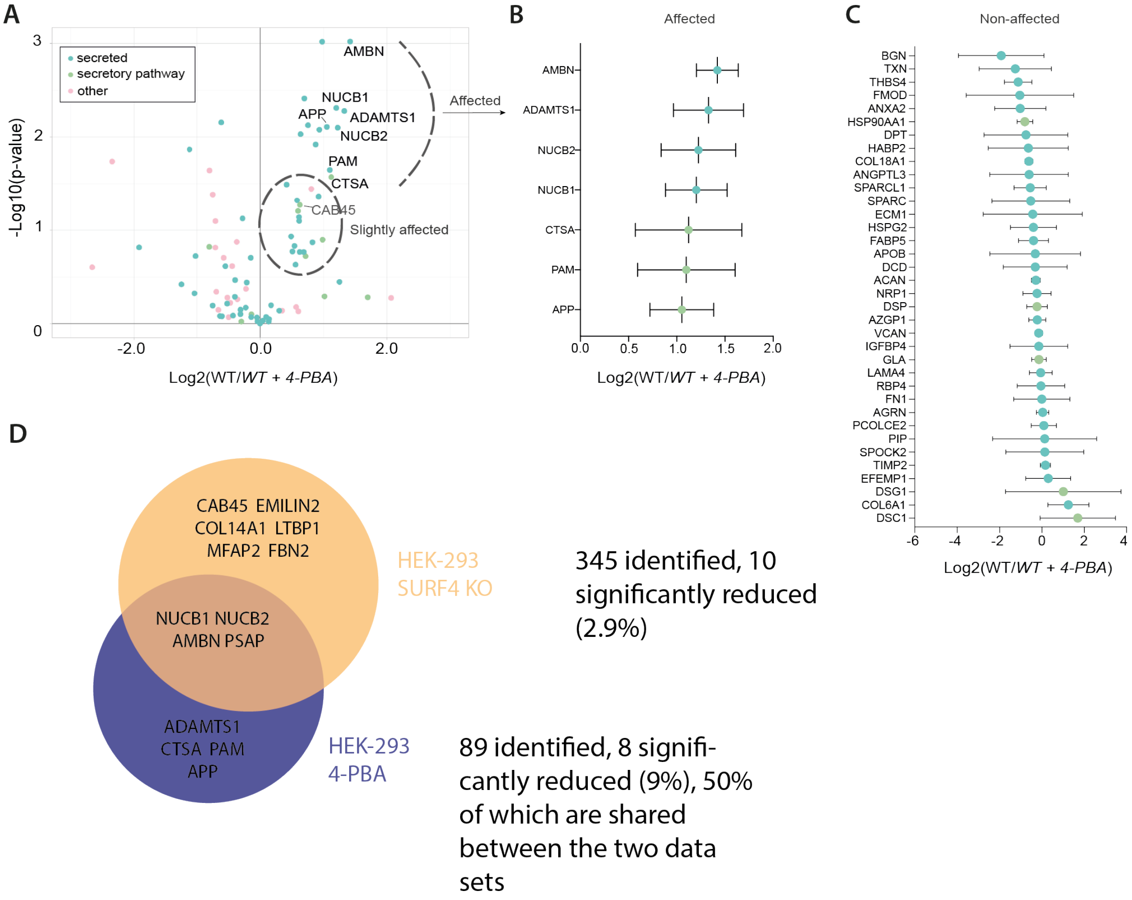

Figure S7: Proteomic analysis of secretomes from 4-PBA treated cells (A) Volcano plot showing changes in protein secretion upon 4-PBA treatment in HEK-293 TREx cells. Proteins were considered affected if Log2 SILAC ratio was >1 and p<0.05 **(B)** Bar graph showing proteins with significantly decreased secretion upon 4-PBA treatment. **(C)** Bar graph showing proteins whose secretion was not significantly affected by 4-PBA treatment. **(D)** Venn diagram showing overlap between SURF4 KO and 4-PBA treated samples. Total proteome pool in each sample is also indicated.

Figure S8

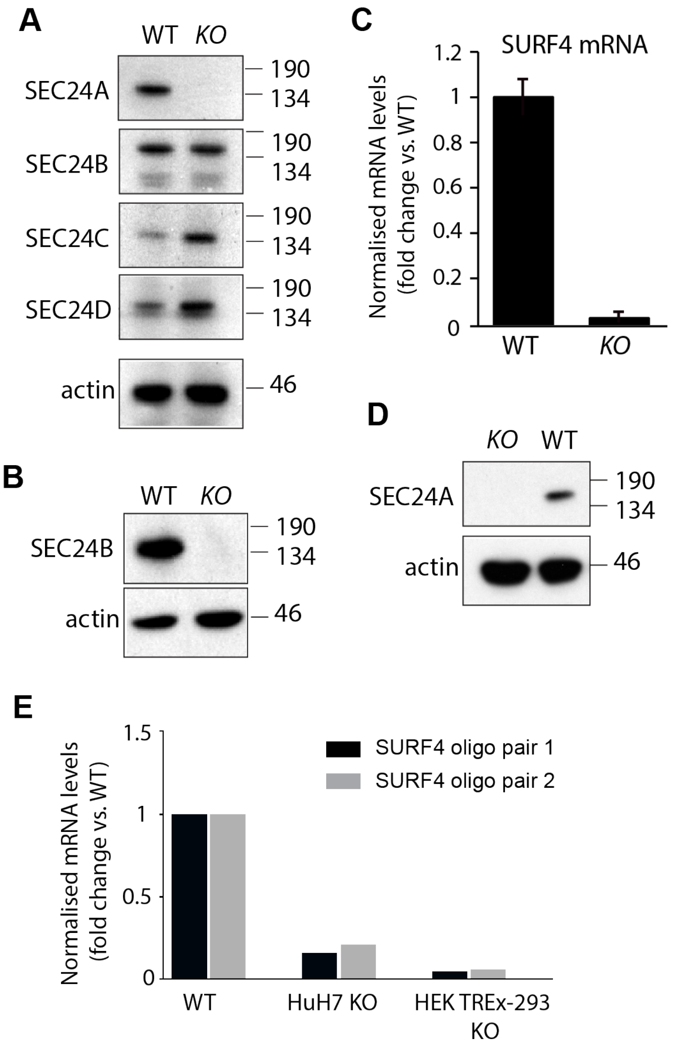

Figure S8: Validation of KO cell lines (A) Western blot analysis of *SEC24A KO* CRISPR/Cas9 edited HEK-293 cells. Stability of Sec24 paralogs was analysed by immunoblotting cell lysates prepared from equal numbers of cells with specific antibodies (α-SEC24A, α-SEC24B, α-SEC24C and α-SEC24D) in WT and *SEC24A KO* cell line. α-actin was used as loading control. **(B)** Western blot analysis of *SEC24B KO* CRISPR/Cas9 edited HEK-293 cells. The presence of Sec24B was analysed by immunoblotting cell lysates prepared from equal numbers of cells with α-SEC24B antibodies. α-actin was used as loading control. **(C)** SURF4 mRNA expression in *SURF4 KO* CRISPR/Cas9 edited HEK-293 cells. Values are presented as mean ± SD of n = 3 independent experiments. **(D)** SURF4 mRNA expression in *SURF4 KO* CRISPR/Cas9 edited HuH7 cells. Values are presented as mean ± SD of n = 3 independent experiments.

Table S1. Plasmids used in this study.

Plasmid	Description	Source
pPCSK9-V5	pcDNA5-FRT-TO-PCSK9-V5	Genescript
pPCSK9-EEE-V5	pcDNA5-FRT-TO-PCSK9 (I154E, P155E, W156E)-V5	This study
pFLAG-Cp	pIRESpuro2b-ssHA-FLAG-Cp	This study
pHA-Cp	pIRESpuro2b-ssHA-HA-Cp	Thor et al., 2009
pLgBiT-SURF4	pcDNA3.1-LgBiT-linker-SURF4	This study
pHaloTag-SmBiT	pcDNA3.1-HaloTag-linker-SmBiT	This study
pSmBiT-SEC24A	pcDNA3.1-SmBiT-linker-SEC24A	This study
pSmBiT-SEC24A V748A	pcDNA3.1-SmBiT-linker-SEC24A V748A	This study
pSmBiT-SEC24A R750,752A	pcDNA3.1-SmBiT-linker-SEC24A R750,752A	This study
pSmBiT-SEC24A Y437A	pcDNA3.1-SmBiT-linker-SEC24A Y437A	This study
pSmBiT-SEC24A Y496A	pcDNA3.1-SmBiT-linker-SEC24A Y496A	This study
pSmBiT-SEC24A R541A	pcDNA3.1-SmBiT-linker-SEC24A R541A	This study
pSmBiT-SEC24A K813A	pcDNA3.1-SmBiT-linker-SEC24A K813A	This study
pNUCB1-HA	pcDNA3.1-NUCB1-HA	Genescript
pNUCB1-EEE-HA	pcDNA3.1-NUCB1(V27E, P28E, L29E)-HA	This study
pCab45-HA	pLPCX-Cab45-HA	von Blume et al., 2012
pCab45-EEE-HA	pLPCX-Cab45 (R37E, P38E, A39E)-HA	This study
pCab45-N40G	pLPCX-Cab45 (N40G)-HA	This study
pFLAG-SURF4	pCDNA3.1-SURF4	Genescript

Dataset S1 (separate file). Mass spectrometry data of secreted proteome from HEK293 cells.

Dataset S2 (separate file). Mass spectrometry data of secreted proteome from HuH7 cells.

Dataset S3 (separate file). Mass spectrometry data of secreted proteome from HEK293 cells treated with 4-PBA for 4 hours.
